# Genetic Analysis of Domestication Parallels in Annual and Perennial Sunflowers (*Helianthus* spp.): Routes to Crop Development

**DOI:** 10.3389/fpls.2020.00834

**Published:** 2020-06-12

**Authors:** Sean R. Asselin, Anita L. Brûlé-Babel, David L. Van Tassel, Douglas J. Cattani

**Affiliations:** ^1^Department of Plant Science, University of Manitoba, Winnipeg, MB, Canada; ^2^Agriculture and Agri-Food Canada, Swift Current Research and Development Centre, Swift Current, SK, Canada; ^3^The Land Institute, Salina, KS, United States

**Keywords:** domestication, oilseeds, perennial grains, comparative genomics, ecosystem services, genotype-by-sequencing, functional diversity, sunflower

## Abstract

Parallels exist between the domestication of new species and the improvement of various crops through selection on traits which favor the sowing, harvest and retention of yield potential and the directed efforts to improve their agronomics, disease resistance and quality characteristics. Common selection pressures may result in the parallel selection of orthologs underlying these traits and homologies between crop species can be exploited by plant breeders to improve germplasm. Perennial grains and oilseeds are a class of proposed crops for improving the diversity and sustainability of agricultural systems. Maximilian sunflower (*Helianthus maximiliani* Schrad.) is a perennial crop wild relative of sunflower (*Helianthus annuus* L.) and a candidate perennial oilseed species. Understanding parallels between cultivated *H. annuus* and *H. maximiliani* may provide new tools for the development of Maximilian sunflower and other wild relatives of sunflower as crops to enhance functional diversity in cropping systems. F_2_ populations of Maximilian sunflower segregating for traits associated with the domestication ideotype of cultivated sunflower including branching architecture, capitulum morphology and flowering time were developed to investigate parallels between *H. maximiliani* and *H. annuus*. Genotype-by-sequencing (GBS) was employed to genotype novel Maximilian sunflower populations and perform quantitative-trait-loci (QTL) analysis. A total of 11 QTL in five regions were identified across 21 linkage groups using 4142 GBS derived single nucleotide polymorphism markers called using the sunflower reference genome as a guide. A major QTL on linkage group 17b, associated with aspects of floral development and apical dominance, was discovered and corresponds with a known domestication QTL hotspot in *H. annuus* and candidate genes were identified. This suggests the potential to exploit orthologs for neo-domestication of *H. maximiliani* for traits such as branching architecture, timing of anthesis, and capitulum size and morphology for the development of a perennial oilseed crop from wild relatives of cultivated sunflower.

## Introduction

Annual crops comprise an estimated 60–80% of global cropland and approximately 75% of calories consumed by humans come from four annual grain crops: maize, wheat, rice, and soybean (Lobell et al., 2011). Targeting perennial species and integrating them into agroecosystems dominated by annual crops has been suggested as a method of enhancing functional diversity (i.e., the number of functionally disparate species), ecosystem function (Isbell et al., 2011), and ultimately, productivity through the introduction of new ecosystem services (Asbjornsen et al., 2014). A number of candidate perennial grain and oilseeds crops have been proposed through both the hybridization with annual crops and the identification of wild species with favorable characteristics for neo-domestication (Wagoner and Schaeffer, 1990; DeHaan et al., 2016).

Maximilian sunflower (*Helianthus maximiliani* Schrad.) is a perennial crop wild relative of cultivated sunflower (*Helianthus annuus*) and is native to much of the interior plains of North America, with a range extending from southern Canada to northern Mexico (Kawakami et al., 2011, 2014; Tetreault et al., 2016). Maximilian sunflower has been used in range seeding mixtures for high quality livestock forage, as a perennial filter strip to reduce agricultural run-off, and as a source of wildlife food and habitat (Dietz et al., 1992; USDA-NRCS, 2017). Maximilian sunflower produces an oil rich seed safe for human consumption primarily composed of linoleic and oleic acids and surveys of wild populations have shown an oil content of 31.1% (Dorrell and Whelan, 1978; Seiler, 1994; Seiler and Brothers, 1999). The natural range of Maximilian sunflower, along with its phenotypic divergence (Kawakami et al., 2011; Asselin et al., 2018), high seed production potential, and documented resistance to known common pathogens of annual sunflower (*H. annuus* L.) such as Sclerotinia rot [*Sclerotinia sclerotiorum* (Lib.) de Bary] (Taski-Ajdukovic et al., 2006; Liu et al., 2011), has attracted attention to the species for the development of a perennial oilseed. Maximilian sunflower is a candidate species for the development of dual-use perennial crop for edible oil, forage and bioenergy applications and is targeted for domestication and improvement (Cox et al., 2002; Van Tassel et al., 2014; Asselin et al., 2018).

Efforts to improve of Maximilian sunflower has, until recently, been focused on its use for conservation and rangeland applications or as a trait donor for *H. annuus*. Two open pollinated commercialized cultivars, “Aztec” and “Prairie Gold” were released by the United States Department of Agriculture Natural Resources Conservation Service (USDA-NRCS) in 1978 (Texas Agricultural Experiment Station, 1979; Dietz et al., 1992; USDA-NRCS, 2017). Aztec was developed for wildlife feed, livestock forage cover, use as a natural hedge, as filter-strips, and as an ornamental landscape plant. Prairie Gold was released for landscape reclamation and wildlife food plantings. Both cultivars were selected for vigor and stand establishment in Oklahoma and Texas (Aztec), or Kansas and further north (Prairie Gold). Agronomic research on Maximilian sunflower as a perennial grain candidate began at The Land Institute in the 1980s (Jackson, 1990). The first breeding program focused on developing Maximilian sunflower as a perennial grain was launched in 2002 at The Land Institute (Cox et al., 2002) with the intention of domesticating the species as a perennial oilseed. Selection for seed size and apical dominance has been effective and following three rounds of recurrent selection, the average seed size was increased by 240% and plants exhibiting a highly restricted branching habit were developed by 2012 (Van Tassel et al., 2014).

Crop domestication shows many parallels between species. Vavilov in the development of the *Law of Homologous Series* first recognized that heritable variation in common traits will occur in different species based on parallel selection pressures (Vavilov, 1922). The *Law of Homologous Series* states that closely related species, genera and families exhibit parallel variation in shared traits. In grains and oilseeds such as sunflower, parallel selection for a more determinate growth habit, larger inflorescence size, and increased grain size has occurred through domestication (Zohary, 2004; Purugganan and Fuller, 2009; Lin et al., 2012). A number of domestication syndrome and quality traits in annual grains are controlled by alleles with large effects and relatively simple genetic control. Domestication has led to the selection of common orthologs such as *sh1* genes which contribute to shattering tolerance in sorghum, rice and maize (Lin et al., 2012); *Q* genes in wheat (*Triticum aestivum* L.) and barley (*Hordeum vulgare* L.) which confer the brittle rachis/free-threshing trait (Simons, 2005); and variations in fatty acid desaturase genes in oilseeds such as canola (*Brassica napus* L.) and sunflower (Peng et al., 2010; Chapman and Burke, 2012). Strong apical dominance and restricted branching were key traits identified for the domestication of *H. annuus* (Burke et al., 2002; Wills and Burke, 2007). Wild type Maximilian sunflower has many similarities to wild type *H. annuus*, which exhibits profuse branching, small achenes, indeterminate flowering and lacks shattering resistance (Burke et al., 2002; Wills and Burke, 2007). Maximilian sunflower individuals with restricted branching, resembling the unbranched ideotype of cultivated sunflower, have been identified in germplasm developed at The Land Institute (Cox et al., 2010; Van Tassel et al., 2014) and offer a unique opportunity to examine parallels in the domestication process in *Helianthus*. *H. annuus* and *H. maximiliani* are both diploid species (2*n* = 17) capable of hybridization and cytogenetic analysis has shown a high proportion of normal bivalents in hybrids, suggestive of high homology between parental chromosomes (Jan, 1997; Binsfeld et al., 2001). As an extension of Vavilov’s *Law of Homologous Series*, knowledge of the domestication and genomic resources available in annual sunflower could be applied as a model for the neo-domestication and improvement of Maximilian sunflower through marker-assisted-selection (MAS) for domestication orthologs.

Genotype-by-sequencing (GBS) has successfully been utilized for species in which heterologous reference genomes are available for single nucleotide polymorphism (SNP) discovery, such as for wild crop relatives of soybean (Chang et al., 2014), wheat (Edae et al., 2016), and sunflower (Baute et al., 2016; Asselin et al., 2018). The goals of this research were to examine parallels between the domestication of cultivated sunflower and the development of Maximilian sunflower as a perennial oilseed crop. This work involved applying GBS to Maximilian sunflower using annual cultivated sunflower as a reference genome and performing quantitative-trait-loci (QTL) analysis in a novel Maximilian sunflower population segregating for apical dominance.

## Materials and Methods

### Development of Maximilian Sunflower With Restricted Branching at the Land Institute

Seed from 96 wild Kansas populations were collected in the autumns of 1999 and 2000. Most of these were roadside populations in areas dominated by native rangeland. Ten plants from each population were transplanted to the field at The Land Institute in 2001 and evaluated for two growing seasons. One hundred and fifty individuals with higher than average seed production were selected and clonally replicated by rhizome division in 2003 for further assessment. Seed yield and seed mass data from the 2004–2005 growing seasons were used to select 20 genotypes for polycrossing. Polycrosses were performed by pollen bulking and subsequent manual pollination in 2006. Polycross seed from each of the 20 genotypes was sprouted in the greenhouse and 112 vigorous seedlings from each maternal family were transplanted to a new field in 2007. Although the purpose of this cycle was originally to estimate trait heritability using the 20 half-sib families, a single unbranched individual was found in 2007. Assuming that the unbranched trait was recessive, we planted 100 open-pollinated (half-sibling) seeds from this genotype in 2008, none of which exhibited restricted branching. Half-sibling plants were allowed to intermate and progeny were evaluated in the 2009 growing season for branching characteristics. Reduced branching individuals reappeared in the progeny, however, many of these plants exhibited reduced seed fertility. Plants with the least amount of branching and normal capitula morphology were polycrossed in 2009 and repeated cycles of visual selection for restricted branching and intermating occurred in 2010 and 2011 to develop seeds stock for populations with restricted branching. In 2012 crosses were performed between plants exhibiting restricted branching to generate full sibling families exhibiting limited to no branching.

### Development of F_2_ Mapping Populations

The mapping populations described in this study were produced through crossing highly-branched wild-type *H. maximiliani* plants from Manitoba to advanced, restricted branching populations developed by The Land Institute (TLI), Salina, Kansas. Manitoban plants were selected as a parent to introduce genetic diversity to alleviate potential inbreeding depression observed in TLI germplasm and to initialize the introduction of the restricted branching trait into an earlier flowering genetic background better suited for northern growing conditions. Maximilian sunflower exhibits clinal variation in life history traits, most notably timing of flowering which occurs in July–August in Manitoban genotypes and September–October in Kansas genotypes (Heiser et al., 1969; Kawakami et al., 2011; Cattani and Van Tassel, personal communication). Full-sib families were developed for mapping traits associated with branching, capitulum size and flowering time. Five restricted branching populations from TLI (“o/i,” “I/P,” “I/H,” “h/L,” and “h/e”) were initially screened under controlled environments. Plants exhibiting a complete restriction of branches, late flowering genetic background and a single large capitulum (30–40 mm in diameter) were selected as a parents for the development of the mapping populations. The Manitoba parents consisted of wild plants collected from roadside sites across southern Manitoba. Manitoban populations, previously described under growth chamber conditions in Asselin et al. (2018), exhibit extensive branching (∼11–15 branches), an early flowering genetic background and a large number (∼12–20) of small capitula which are ∼7–21 mm in diameter. A series of crosses were produced and F_1_ seed derived from a cross between an individual from the TLI population “o/i” and a wild Manitoban accession collected near Brunkild, Manitoba (49.49°N, 97.57W) were grown for sib-mating. The F_1_ plants were clonally propagated from rhizome cuttings to produce materials for sib-crossing. Three F_1_ individuals (herein denoted as F1A, F1B, and F1C) were intercrossed through reciprocal sib-mating to generate two F_2_ populations which segregated for branching, herein denoted as crosses AB (F1A/F1B) and BC (F1B/F1C).

### Phenotypic Evaluation

#### Plant Propagation

Seeds of the F_2_ were surface sterilized using a 70% ethanol solution for 10 min, allowed to air dry, and seed coats were then manually removed to break seed dormancy. Seeds were placed in 9 cm petri dishes on filter paper moistened with distilled water containing a 0.1% solution of plant preservative mixture (PPM^TM^, Plant Cell Technology, Washington, DC, United States). Petri plates were placed in the dark at room temperature for 48 h to germinate. Germinated seeds were transplanted once radicles had emerged. All seedlings were started in 6-well 122.9 mL volume seeding trays filled with Sunshine #4^TM^ soilless potting mix (SunGro Horticulture Ltd., Agawam, MA, United States) and transferred to 1 L pots containing a 2:2:1 ratio of soil:sand:peat by volume once they had reached the three to four true-leaf stage. Plants were grown in a growth chamber under a 23°C 16-h day/18°C 8-h night cycle. Plant positions were assigned randomly and rotated on a weekly basis to account for potential differences in airflow, humidity, and light intensity across benches within the growth chamber. Phenotypic traits measured can be broadly classified as capitulum size traits, traits relating to anthesis, traits associated with branching architecture, traits related to total plant size. Seed yield and size traits were not examined in this study directly due to the constraints of the testing location and ability to cross-pollinate all F_2_ progeny examined.

#### Trait Evaluation

Sixteen traits were phenotyped on 163 and 177 individuals from the AB and BC crosses, respectively, for a total 340 F_2_ plants (Table 1). As the parental and F_1_ individuals underwent photoperiod induction (resulting in skewed phenotypes), complete phenotypic analysis was conducted solely on the F_2_ generation. Phenotyping of the appearance of reproductive buds and date of the first capitulum to reach anthesis through fifth capitulum to reach anthesis were conducted three times a week. As plants segregated for flowering time, the date of fifth anthesis was employed as a common physiological milestone at which all other traits were assessed. All nodes were counted starting at the cotyledonary node moving upwards along the main stem. Branches were measured as the total number of branches on the main stem greater than 2 cm in length. The highest branching node, a measurement of apical branching, was measured as the total number of unbranched nodes which were found above the highest branch on the apical portion of the stem. The lowest branching node, a measurement of basal branching, was measured as the number of unbranched nodes on the basal region of the stem between the cotyledonary node and lowest branch. Capitulum morphology was characterized to infer potential traits associated with shattering resistance. Shattering in wild *H. annuus* is in part due to the continued growth of the capitulum resulting in a convex shape (increased depth: width ratio). Domesticated *H. annuus* exhibits a low depth: width ratio, resulting in a flatter capitulum, less prone to shattering (Burke et al., 2002). As plants were not evaluated for seed traits with the depth: width ratio employed as a proxy for this trait.

**TABLE 1:** Descriptions of 16 phenotypic traits measured under growth chamber conditions for 340 *H. maximiliani* F_2_ plants following a 23°C 16-h day/18°C 8-h night cycle.

Capitulum traits	Description
Diameter of capitulum 1–5 (ACW)	Average diameter of the first five capitulum to reach anthesis in mm
Depth of capitulum 1–5 (ACD)	Average depth of the first five capitula to reach anthesis in mm
Depth:width ratio (DWR)	Average capitulum depth/width in mm
Size of central capitulum (CCW)	Size of central capitulum in mm
Total capitula (CC)	Total number of capitula above a minimum size of 2 mm
**Branching traits**	
Length of branches 1–5 (BL)	Average length of the first five apical branches above 2 cm in in length measured in cm
Highest branching node (HBN)	Measurement of the highest branching point on the main stem. Measured as the number nodes on the main stem above the highest branch with a branch length greater than 2 cm in length
Lowest branching node (LBN)	Measurement of the lowest branching point on the main stem. Measured as the number of basal nodes of the main stem below the lowest branch with a branch length greater than 2 cm
Branches (B)	Number of nodes bearing branches greater than 2 cm in length
**Plant size traits**	
Total nodes (TN)	Total plant nodes apparent on the main stem
Stem diameter (SD)	Stem diameter measured at the first basal node in mm at maturity
Plant height (PH)	Plant height in cm
Length of leaf 1–5 at maturity (LL)	Length of first five leaves below the central capitulum in cm
**Anthesis traits**	
Emergence of apical buds (RB)	Number of days to the first appearance of reproductive buds
First anthesis (FA)	Number of days to first anthesis
Average anthesis for the first 5 capitula (AA)	Average days to first anthesis for the first five capitula

Data were analyzed for normality using the SAS UNIVARIATE procedure to assure data meet assumptions of normality for downstream analysis. Principal component analysis was conducted to explore trait relationships on centered and scaled data using the R function *PRCOMP*. The number of principal components to retain was determined by examining a breakdown of eigenvectors following Cattell’s rule (Cattell, 1966) and Horn’s parallel analysis (Horn, 1965) was run using 1000 permutations in the R package *paran* (Dinno, 2012) and adjusted eigenvectors >1 were retained for analysis.

### DNA Extraction

Leaf tissues from all plants grown under growth chamber conditions were sampled for DNA extraction. Samples from each plant were labeled with an identity number and frozen in liquid N within 1 h of collection prior to lyophilization and storage at room temperature. Genomic DNA was extracted from the parental, F_1_ and a subset consisting of 190 F_2_ individuals, consisting of 78 individuals from the AB cross and 112 individuals from the BC cross. A modified single-tube cetyltrimethylammonium bromide (CTAB) extraction protocol (Doyle, 1987; Li et al., 2007) was employed with 1 μL of 10 mg mL^–1^ solution of proteinase K (Promega) added to the initial CTAB incubation step. DNA quantity was determined using a dsDNA broad Range Fluorescence Assay kit and a Qubit 2.0 Fluorometer (Life Technologies) following the manufacturers’ instructions for 1 μL sample sizes. DNA quality was assessed using 260/280 and 260/230 nm wavelength absorbance ratios measured using a Nanodrop 1000 spectrophotometer (Thermo Fischer Scientific). Samples that fell below a minimum DNA concentration of 50 μg/μL or absorbance ratios below 1.7 for either 260/230 or 260/280 nm were discarded and extractions were repeated to meet quality requirements.

### Genotype-by-Sequencing and SNP Calling

One-hundred and ninety-five DNA samples consisting of the Manitoba and Kansas parentals, F_1_ parents and 190 F_2_ plants were submitted to Data2bio LLC (Ames, IA, United States) for GBS and SNPs calling. A tunable genotype-by-sequencing (tGBS) protocol was employed, which differs from conventional GBS through the use of a greater selective genome reduction procedure (Ott et al., 2017). Fewer sites are sequenced in tGBS relative to the conventional GBS, resulting in greater read depth. The advantage of this approach is that greater depth of coverage allows for more effective calling of heterozygous genotypes and reduces false homozygote calls, leading to a lower level of missing data per site.

Sequencing via tGBS was carried out using a three selective base (TGT) protocol as described in Ott et al. (2017) for genome reduction. Fragments were sequenced via eight runs on an Ion Proton^TM^ sequencer (Thermo Fisher Scientific, Waltham, MA, United States). The conservative three selective base approach was chosen as *H. maximiliani* is an obligate outcrossing species and exhibit a high degree of heterozygosity (Asselin et al., 2018). Trimmed reads were aligned to the cultivated *H. annuus* reference genome, HA412.v.1.1.bronze.20142015,^1^ using GSNAP (Wu and Nacu, 2010) with polymorphisms within the first and last 3 bp for the read being ignored (Ott et al., 2017). Polymorphisms with base calls with a PHRED score below 20 were removed from the analyses. The SNPs were called as homozygous if the most common allele was supported by a minimum of 80% aligned reads, while heterozygous SNPs were called if the two most common alleles were supported by a minimum of 30% of all aligned reads and the sum of the two most common alleles accounted for a minimum of 80% of the aligned reads. Single nucleotide polymorphisms that could be genotyped in a minimum of half of the samples, that contained an allele number of 2, a minor allele frequency >1% and a heterozygous rate of <70% were maintained for downstream analysis. Imputation was conducted separately on the AB and BC crosses using Beagle4.1 (Browning and Browning, 2016) using default parameters.

### Linkage Map Construction

The AB and BC crosses were examined jointly as a single population employing markers segregating in the same fashion between the two crosses for analysis in Joinmap 5.0 (Van Ooijen, 2018). Common segregation patterns between the crosses were determined using the F_1_ genotypes and observed segregation patterns in the F_2_ generation to allow joint analysis. Markers were coded as three possible segregation types (<lmxll>, <nnxnp>, and <hkxhk>) corresponding to backcross-like or F_2_-like segregation patterns. Homozygous allele states accounting for less than 10% of the observed total calls were considered likely genotyping errors and set to missing data prior to analysis. Markers classes in which either the parental genotype could not be accurately determined or were non-informative were removed. The remaining markers were tested for distorted segregation using a chi-square goodness-of-fit test through the test segregation function of Joinmap at *p* < 0.05 for downstream analysis. Due to the presence of a high degree of segregation distortion, markers exhibiting distortion were retained within the dataset and initially grouped based upon their assigned linkage groups in the cultivated *H. annuus* HA412.v.1.1.bronze.20142015 reference genome for ordering. Segregation distortion has been reported as having little effect on marker order (Zhang et al., 2010), but erroneously called genotypes, which may result in the appearance of highly-distorted markers, may cause pseudo-linkages between biologically unlinked sequences (Ronin et al., 2010).

Linkage map construction was conducted using the cross-pollinated (CP) analysis option in Joinmap 5.0. Identical markers were removed and reference genome positions were utilized in initial maps using the “start order” option to position markers and linkage groups were numbered based upon their reference genome analog. Markers were ordered using the regression mapping algorithm with map distances in cM being calculated using Kosambi’s genetic distance. Markers which produced negative map distances were excluded from the map and maps were recalculated. In instances were insufficient linkage was present within a linkage group markers were split into separate groups (i.e., LG 3a and 3b) for analysis to produce the final map.

### QTL Analysis

Restricted multiple-qtl model (rMQM) analysis was conducted using MapQTL 6.0. As a subset of the genotyped F_2_ plants exhibited an abnormal “die-back” of the central capitulum, 15 plants were removed from the dataset, leaving 175 individuals for QTL analysis. An initial analysis was conducted to identify putative QTL and possible cofactors for rMQM using the interval mapping procedure at 1 cM intervals. A non-parametric one-way analysis of variance Kruskal–Wallis (K–W) rank-sum test was also conducted. The K–W test method does not require the incorporation of a genetic map, providing marker-trait associations independently of neighboring markers and was used to confirm putative co-factors and QTLs. To account for multiple testing, a stringent alpha of 0.005 was used to reduce potential spurious associations as suggested by Van Ooijen (2018). Possible rMQM cofactors were limited to no more than two per linkage group per trait and an automatic cofactor selection procedure in MapQTL 6.0 was performed using default settings. Subsequent rounds of rMQM mapping were performed for each trait until co-factors stabilized. To account for multiple testing and control of family-wise-error rate for type I errors, whole-genome LOD thresholds were calculated for each trait independently (Churchill and Doerge, 1994). Appropriate genome-wide LOD threshold values were estimated using 1000 permutations and an alpha threshold of *p* < 0.05. A genome-wide LOD threshold score of *p* < 0.05 was employed to declare significant QTL. Confidence intervals were calculated using the two-LOD drop-off method which approximates roughly a 95% confidence interval of QTL position (Lander and Botstein, 1989). Strongly overlapping/pleiotropic QTL between two or more traits were given a common name to designate the QTL region.

To infer candidate genes and provide functional annotation of the candidate SNPs generated from the analyses, *H. maximiliani* SNPs falling within the two-LOD QTL confidence interval were compared with the cultivated *H. annuus* reference genome HA412.v.1.1.bronze.20142015 assembly using Jbrowse (Skinner et al., 2009), available via the HeliaGene bioinformatics portal (Carrere et al., 2008). Significant SNPs found within a 1kb distance were considered a common entry for annotation. SNPs were compared to their positions within the *H. annuus* reference genome and a search was conducted for possible candidate genes within 100 kb of each entry. To annotate the presence of putative orthologs, a literature search of sunflower domestication studies was conducted to determine if QTL controlling similar traits have previously been reported on the same linkage groups in *H. annuus*.

## Results

### Phenotypic Analysis

The initial TLI populations screened for restricted branching exhibited either a single capitulum and a complete lack of branching, or a complete lack of mid-plant and basal branches and a small number (2–3) short apical branches bearing capitula. It has been suggested that branching restriction in Maximilian sunflower exhibits a leaky phenotype, and this observation is consistent with previous observations from restricted branching populations (Van Tassel, personal communication). The F_1_ plants were uniform in appearance, and exhibited a branched phenotype consistent with that of wild Manitoban populations. The range of phenotypes observed amongst both crosses was extensive in the F_2_ generation and the phenotypic distributions are suggestive of quantitative genetic control (Table 2). The unbranched phenotype observed in the TLI parent was not recovered in the F_1_ or F_2_ generations although a number of plants exhibiting highly restricted branching were observed in the F_2_ generation. Branch number ranged from one to 21 branches and the percentage of branch bearing nodes ranged from 2.38 to 48.97%. Timing of first anthesis ranged from 61 to 198 days. Plant height ranged from 90to 192 cm and diameter of the central capitulum ranged from 6.2to 25.6 mm. The total number of capitulum exhibited the highest coefficient of variation relative to other traits examined, ranging from 4 to 126 capitula per plant likely due to the presence of secondary and tertiary branches.

**TABLE 2:** Phenotypic mean, standard error of the mean (SEM) and coefficient of variation (CV) and other summary statistics of 340 F_2_ *H. maximiliani* plants phenotyped for 21 traits under growth chamber conditions following a 23°C 16-h day/18°C 8-h night cycle.

Capitulum traits	Mean	SEM	Skewness	Kurtosis	Range	CV (%)
Mean diameter of capitula 1–5 (mm)	12.6	0.13	0.05	-0.41	14.18	20
Mean depth of capitula 1–5 (mm)	11.8	0.09	0.07	-0.08	12.04	15
Capitulum depth:width ratio	0.95	0.005	1.06	4.11	0.9	11
Size of central capitulum (mm)	13.89	0.18	0.35	-0.07	19.4	24
Total capitula count	39.64	1.21	1.36	1.83	122	56
**Branching**						
Average length of branches 1–5 (cm)	29.41	0.52	0.23	-0.04	57.8	33
Highest branching node	5.6	0.12	1	2.33	15	40
Lowest branching node	36.28	0.59	0.21	0.83	68	30
Total Branches	8.31	2.78	1.07	2.07	20	33
**Plant size**						
Total nodes	50.96	0.58	0.59	0.84	67	21
Stem diameter (mm)	9.53	0.07	0.26	0.51	8.5	14
Plant height (cm)	142.11	1.11	-0.07	-0.63	102	15
Length of leaf 1–5 at maturity (cm)	12.53	0.21	0.21	-0.22	22.1	31
**Anthesis**						
Emergence of reproductive buds (days)	67.44	0.78	-0.02	-0.03	82	21
First anthesis (days)	118.37	1.34	0.66	0.46	137	21
Average anthesis for the first 5 capitula (days)	133	1.33	0.34	-0.05	134.8	18

**FIGURE 1:**
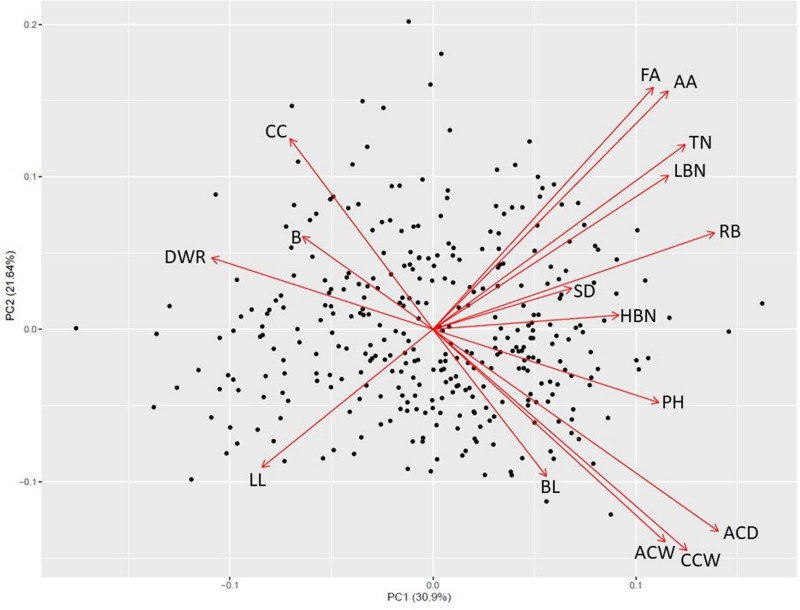
First and second principal component axes for 340 Maximilian sunflower individuals segregating for apical dominance. FA, First anthesis; AA, average date of anthesis; TN, total nodes; LBN, lowest branching node; RB, reproductive budding; SD, stem diameter; HBN, highest branching node; PH, plant height; ACD, average capitula depth; CCW, central capitulum width; ACW, average capitula width; BL, branch length; LL, leaf length; DWR, capitulum depth:width ratio; B, branches; CC, total capitula count.

Principal component analysis revealed four significant components and patterns suggestive of a resource allocation trade-off relating to the degree of apical dominance (Figure 1 and Supplementary Table S1). Along the first two principal components axes a negative relationship between capitulum size traits (greater apical dominance) and the total number of branches and capitula (reduced apical dominance) is apparent. The first component (PC1) which explained 30.9% of the phenotypic variance appears to be dominated by trait differences in the parental materials, with positive loadings for later anthesis and increased capitulum size and negative loadings for capitulum and branch number. The second component (PC2), explaining 21.64% of the phenotypic variance revealed patterns between timing and anthesis and capitulum size traits which contradict the first component, suggesting these traits are not strongly associated and may represent recombination between parental types. The third and fourth components (Supplementary Figure S1), explaining 9.84 and 8.36% of the phenotypic variance respectively were dominated by branching characteristics (PC3) and other aspects of apical dominance (highest branching node, average branch length and capitulum count) as well as stem diameter (PC4).

### SNP Calling and Linkage Map Development Using the Homeologous *H. annuus* Genome as a Reference Guide

A total of 6094 polymorphic SNPs were aligned to the homeologous reference genome of *H. annuus* for QTL identification spanning all linkage groups. Following filtering 5323 SNPs were considered for joint linkage map development and QTL analysis. Due to insufficient linkage more than the expected 17 linkage groups of *H. maximiliani* were recovered, linkage groups 3, 5, 13, and 17 were split into separate groups resulting in 21 linkage groups (Supplementary Figure S2). To date, this is the first reported linkage map of Maximilian sunflower and spans a total of 3159.29 cM of the Maximilian sunflower genome, with an average marker interval of 1.31 cM. A total of 4142 markers were incorporated into the map, with 42–300 markers per linkage group (Supplementary Table S2).

### QTL Analysis and Identification of Putative Candidate Genes Associated With Domestication

The Genome-wide QTL scans detected 11 significant QTL that were supported by both the rMQM and K-W analysis which corresponded to five genomic regions. A major co-localized QTL (herein referred to as *ultra1*) was detected on linkage group 17b explaining 12.1–21.5% of the variation in traits associated with timing of anthesis (timing of reproductive budding, first anthesis and average date of anthesis) as aspects of apical dominance (number of branches, average leaf length, average capitulum size, and average capitulum depth) (Table 3). Further QTL for timing of reproductive budding were found on linkage groups 6 (*rb1*) and 15 (*rb2*), and total capitulum count on linkage groups 2 (*cc1*) and 3b (*cc2*).

**TABLE 3:** Significant genome-wide QTL in the tested Maximilian sunflower populations (*p* < 0.05).

QTL	Trait	Nearest Markers	Linkage group	Peak (cM)	CI^1^ (cM)	%Var^2^	LOD^3^
*ultra1*	Timing of reproductive budding	Ha17_43720802	17b	118.57	116.76–119.31	12.1	6.22
	First anthesis	Ha17_43720799	17b	117.14	116.72–119.31	18.8	5.54
	Average date of anthesis	Ha17_43720802	17b	118.57	116.76–119.31	20.7	7.51
	Total branches	Ha17_38911771	17b	118.57	117.14–123.31	18.8	6.71
	Average leaf length	Ha17_43720799	17b	117.14	113.47–119.31	15	6.5
	Average capitulum width	Ha17_43720799	17b	117.14	111.47–119.31	17.4	6.17
	Average capitulum depth	Ha17_43720802	17b	117.57	112.47–119.31	21.5	7.34
*rb1*	Timing of reproductive budding	Ha6_80827033	6	95.15	92.57–99.08	11.6	7.39
*rb2*	Timing of reproductive budding	Ha15_4372179	15	12.5	10.50–13.17	11.7	7.66
*cc1*	Total capitulum count	Ha2_149340796	2	68.63	65.64–70.91	10	6.43
*cc2*	Total capitulum count	Ha3_54508649	3b	76.54	76.14–77.54	12.4	6.32

*^1^CI, Two-LOD confidence interval as approximated using the method of Lander and Botstein, 1989, ^2^%Var, Percent of variation in which the QTL explains, ^3^LOD, likelihood of odds.*

To identify putative genes associated with the domestication phenotype of *H. annuus* 100 kb flanking regions surrounding SNPs within the two-LOD QTL support intervals were manually examined using the sunflower genome JBrowse (INRA Sunflower Bioinformatics Resources, 2014). None of the QTL associated SNPs were found within gene sequences but several were within close proximity to annotated genes (Supplementary Table S3).

The SNPs associated with the QTL *ultra1* on linkage group 17b of *H. maximiliani* correspond to a gene-rich region of the *H. annuus* reference genome. A variety of putative functions were found within this region relating to hormone response and regulation and meristem development. Nearby genes in the region of *ultra1* include plant GH3 auxin-responsive promotors (IPR004993) and a ULTRAPETALA developmental regulator (IPR020533) (Supplementary Table S3) which is known as a key negative regulator of cell accumulation in shoot and floral meristems in *Arabidopsis thaliana* (Fletcher, 2001; Carles et al., 2004). Genes encoding Cytochrome P450 (IPR001128) and F-box domain proteins (IPR001810), both of which are candidate genes for branching in *H. annuus* (Mandel et al., 2013; Nambeesan et al., 2015), were also identified.

Interestingly, the SNPs associated with *ultra1* are approximately 200 kb downstream to a region containing a number of potentially influential genes associated with AUX/IAA proteins (IPR003311), Auxin response factors (IPR010525) and self-incompatibility S1 proteins (IPR010264) suggesting potential genetic linkage between genes associated with the domestication phenotype of *H. annuus* and self-compatibility as noted in previous studies (Burke et al., 2002; Gandhi et al., 2005; Tang et al., 2006).

The SNPs associated with *cc1* were found to be in proximity to genes which code proteins related to cell proliferation, differentiation and development (ARID/BRIGHT DNA-binding domain, IPR001606) and plant hormone production and regulation (AUX/IAA protein, IPR003311; Auxin response factor, IPR015300; Terpenoid synthase, IPR0089494 and Cytokinin riboside 5′-monophospahte phophoribohydrolase LOG, IPR005269). The annotations for *cc2* included a fibroblast growth factor receptor (IPR016248). While SNPs associated with *rb1* were not found to be within 100 kb of putative candidate genes, these SNPs flank a genomic region containing a phytochrome domain (IPR001294) which is associated with plant light response, the control of photoperiodism and flowering. The presence of a flowering-time QTL on this linkage group is also in line with previous studies which have identified a cluster of paralogous *HaFT* genes on LG 6 which are major contributors to photoperiod sensitivity in *H. annuus* (Blackman et al., 2011). Annotations for *rb2* which corresponds to LG 15 of *H. annuus* did not yield obvious functional candidates within close proximity to the associated SNPs though a QTL for flowering is known on this linkage group in wild X landrace crosses of *H. annuus* (Wills and Burke, 2007; Blackman et al., 2011).

## Discussion

### Genetic Parallels With *H. annuus* and Putative S-Locus Linkage

The release of the sunflower reference genome has enhanced the ability to generate SNP libraries for analyzing both natural and segregating populations of cultivated sunflower as well as their wild crop relatives. The use of the sunflower reference genome greatly assisted in the development of a genetic map of Maximilian sunflower and in the identification of candidate genes.

Several promising candidate genes within 100 kb of *ultra1* were identified in this study, one of which is documented in *Arabidopsis* to influence the phenotypic traits associated with *ultra1. ULTRAPETALA* (*Ult1*) has been shown to regulate a number of development processes including the timing of vegetative to floral transition, maintenance of the shoot apical meristem and termination of floral meristem development in *A. thaliana* (Fletcher, 2001; Carles et al., 2004). This is in line with the associations between *ultra1* and timing of anthesis, overall capitulum size and branching in the present study, which presents a strong case for a causative candidate gene. Plants that carry a *Utl1* loss-of-function mutation show restricted branching and an increase in floral meristem size similar to the Kansas populations utilized in this study.

Another promising candidate gene in proximity to *ultra1* is a GH3 auxin-responsive promotor. The role of auxins, cytokinins, and strigolactones in the control of shoot branching and maintenance of apical dominance is well-documented (Bennett et al., 2006; Shimizu-Sato et al., 2009). Several candidate genes have been identified in sunflower domestication studies relating to the production, regulation, and transport of these hormones (Nambeesan et al., 2015).

Additional candidates underlying *ultra1* include cytochrome P450 and F-box proteins. The cytochrome P450 and F-box gene families include several well-known genes associated with the control of axillary meristem initiation and growth such as the more axillary growth (*MAX*) genes of *A. thaliana* (Stirnberg et al., 2002). One of these genes, *MAX2* encodes an F-box protein which has been shown to co-localize with a branching QTL on LG 17 of *H. annuus* (Mandel et al., 2013).

An interesting parallel between *H. maximiliani* and *H. annuus* was observed in the QTL analysis. The QTL *ultra1* on LG 17b corresponds to a region in the *H. annuus* reference genome which harbors annotations for a number of *S*-locus associated proteins, suggesting potential linkage between *ultra1* and self-compatibility in *H. maximiliani*. Previous studies looking at wild X crop crosses in *H. annuus* have observed a preponderance for crop alleles on this linkage group and a clustering of upwards of 20 domestication and post-domestication QTL in tight linkage with the *S-*locus on LG 17 (Burke et al., 2002; Gandhi et al., 2005; Tang et al., 2006).

The role of *ULTRAPETALA* (*Ult1*) genes in sunflower has yet to be explored but several studies have identified domestication QTL in tight linkage with the *S*-locus on LG 17 of *H. annuus.* Burke et al. (2002) observed a clustering of QTL for flowering time, stem diameter, capitulum number, number of capitula/branch, plant height, number of leaves, peduncle length, achene weight, shattering and total number of selfed seeds on LG 17. Gandhi et al. (2005) subsequently mapped the *S*-locus to the same region on LG 17. Tang et al. (2006) observed several overlapping QTL for seed size and seed oil concertation in close proximity to the *S*-locus on LG 17. Wills and Burke (2007) also observed overlapping QTL for capitulum diameter, branch number, ray number, total capitula number and number of selfed seeds again in the same region on LG 17.

The presence of an *S*-locus on LG 17b in Maximilian sunflower may impact breeding efforts and is one explanation that helps explain the reduced fertility observed in the initial Kansas populations selected for apical dominance. This observation warrants further investigation as other forms of inbreeding depression reducing fertility cannot be ruled out at this stage. Tight linkage between domestication QTL such as *ultra1* and an *S*-locus could impose restrictions on domestication if linkage cannot be broken. Stringent selection for a given variant of a *ultra1* allele may result in a genetic bottleneck and reduced *S*-locus allelic diversity. Limited genetic diversity in breeding pools of Maximilian sunflower may limit seed yield through the lack of compatibility in pollen sources, reducing to ability to generate novel germplasm through directed crosses. Fine-scale genetic mapping of *ultra1* and the *S*-locus and development of further SNP markers in Maximilian sunflower would provide an efficient way to break the putative genetic linkage between these loci through the identification of recombinant progeny carrying different *S*-locus alleles.

### Phenotypic Trade-Offs in the Context of Neo-Domestication

A trade-off between capitulum size and capitulum number was observed along both the first and second principal component axis in the F_2_ populations and is suggestive of a putative resource allocation trade-off between capitulum size and capitulum number. In *H. annuus*, unbranched biotypes have larger capitula and seeds than their branched counterparts.

The presence of a major QTL such as *ultra1* influencing flowering time, branching and capitulum morphology would explain the first principal component of the PCA which is dominated by type differences observed in the parental materials. The presence of *ultra1* helps explain the phenotypic correlations between traits such as later flowering and increased capitulum width and depth. Interestingly, the second principal component shows the opposite relationship between these traits suggesting this trait correlation is not absolute.

While selection for a single large capitulum is a defining domestication syndrome characteristic of cultivated annual sunflower, the path perennial oilseeds will take through domestication will ultimately depends on the how standing genetic variation will contribute to yield. In sunflower, branching not only interacts with capitulum size and total capitula number but also seed weight and oil content amongst other traits (Fick et al., 1974; Dedio, 1980; Tang et al., 2006; Bachlava et al., 2010).

While there are inherent benefits to restricting branching, such as the facilitation of mechanical harvest and uniform maturity, restricting branching may also limit yield potential if selected for too stringently. Branched Maximilian sunflower plants are capable of producing a large number of capitula per stem^–1^ (>100) while completely unbranched plants exhibited a single, central capitulu*m*. The relationship between capitulum size and capitula number in Maximilian sunflower is not a 1:1 ratio. Plants with restricted branching have the tendency to exhibit larger capitula, though this increase in capitulum size does not appear to fully compensate for the loss of capitulum number and it has been suggested that this may limit seed yield (Van Tassel et al., 2014; DeHaan et al., 2016). Selection for a single central capitulum, akin to the domestication ideotype of cultivated sunflower, could decrease seed production in *H. maximiliani* if the loss of capitula through restricted branching is not compensated for by a reciprocal increase in capitulum size. Conversely, the single capitulum ideotype may be better suited for polyculture use as opposed to a monoculture, as the loss of yield associated with an unbranched stem may be compensated for by diversification within the field (Picasso et al., 2008; Picasso et al., 2011).

*Helianthus annuus* was initially domesticated for its edible seed, pigments and medicinal compounds (Heiser, 1951) with selection for oil content and composition occurring more recently (Burke et al., 2005; Chapman and Burke, 2012). Therefore, the defining characteristics of its domestication may not necessarily apply to the neo-domestication of Maximilian sunflower as an oilseed crop. Selection for a Maximilian sunflower ideotype as an oilseed may parallel other small-seeded *Compositae* oilseeds such as safflower (*Carthamus tinctorius*) and noug (*Guizotia abyssinica*) both of which exhibit contrasting domestication syndromes to annual sunflower (Pearl et al., 2014; Dempewolf et al., 2015), having retained a branched architecture and selection for greater seed production. Similarly, successful oilseed crops in Western Canada such as canola (*B. napus* L.) retain a branched architecture, small seed size, and are still prone to considerable shattering losses under certain conditions (Gulden et al., 2003; Cavalieri et al., 2016). Despite these characteristics, these crops are capable of sustaining economic yields supporting their use as crops. Increased branching and seed number may constrain seed size, in *H. annuus* and smaller seeds tend to bear a higher concentration of oil. Estimates of 6.7–8.5% greater oil concentration in the seed of branched individuals relative to unbranched individuals in segregating populations have been observed, presumably due to a thinner pericarp (Tang et al., 2006; Bachlava et al., 2010). Therefore, increasing branching and seed number may prove beneficial in increasing oil yield per unit area.

Though selection for restricted branching may limit the total production of capitula, and by extension yield potential on a single plant basis, branching restriction may enhance yield on a per unit area. It has been observed that in species such as safflower lower order branches are often infertile and that in general, plants that produced many branches produced smaller capitula, fewer florets per branch, and smaller seeds in smaller quantities (Bidgoli et al., 2006). Branching restriction may enhance harvestable yield indirectly through greater synchronicity of flowering, maturity of capitula and through changes in capitulum morphology, both of which may alleviate shattering losses. In the present study, the capitulum depth:width ratio appears to decrease with branching restriction and the increase in capitulum size. Plants with a wider, flatter capitulum, more akin to those of domesticated sunflower may exhibit reduced shattering losses if these patterns are common between species. Furthermore, it may be possible to compensate for the loss of secondary capitula through increasing stem density on a per unit area, either through selection for a greater number of stems per plant or sowing higher density plantings coupled with tolerance to crowding. Ultimately, increasing the harvest index of Maximilian sunflower through conventional as well as marker-assisted breeding approaches appears to be possible through multiple pathways and further investigation into these factors under realistic field conditions is required to define the optimal ideotype for production.

In summary, we were successful in identifying QTL for important traits associated with domestication in Maximilian sunflower and identified candidate genes using the existing genomic resources of cultivated sunflower. Results presented in this study have implications for the defining an appropriate ideotype for perennial oilseed crops in the *Compositae* and the development of genomic resources in crop wild relatives. Further work is needed to compare contrasting Maximilian sunflower ideotypes and characterization is required to verify the presence of putative *S*-locus linkage and the development of markers for this and other domestication QTL. Future work will focus on the field based evaluation of yield components in proposed Maximilian sunflower ideotypes and the development of a *de novo* genetic map for Maximilian sunflower and fine mapping of genomic regions surrounding *ultra1.* Understanding both the genetic and agronomic parallels of domestication will play an important role in the neo-domestication of Maximilian sunflower and related species.

## Data Availability Statement

The SNP dataset generated in this study is available in Dryad digital repository (doi: 10.5061/dryad.tdz08kpwt). Any other datasets and germplasm used and/or analyzed during the current study will be made available through contact with the corresponding author on reasonable request.

## Author Contributions

SA generated the mapping populations, conducted the greenhouse and laboratory work, analyzed the data, and wrote the manuscript as a graduate student. DC and AB-B provided laboratory resources and supervision of experimental design and aided in the editing of the manuscript. DV developed the initial restricted branching populations and provided germplasm and feedback on the manuscript. All authors have reviewed and agreed on the contents of the manuscript.

## Conflict of Interest

The authors declare that the research was conducted in the absence of any commercial or financial relationships that could be construed as a potential conflict of interest.

## Footnotes

1 www.sunflowergenome.org
