# Supplementary material for: Genetic Analysis of Domestication Parallels in Annual and Perennial Sunflowers (Helianthus spp.): Routes to Crop Development
Source: Front Plant Sci. 2020 Jun 12;11:834. doi: 10.3389/fpls.2020.00834 (PMC7304338; doi:10.3389/fpls.2020.00834)
Supplement: Supplementary file 1 [file Data_Sheet_1.PDF]

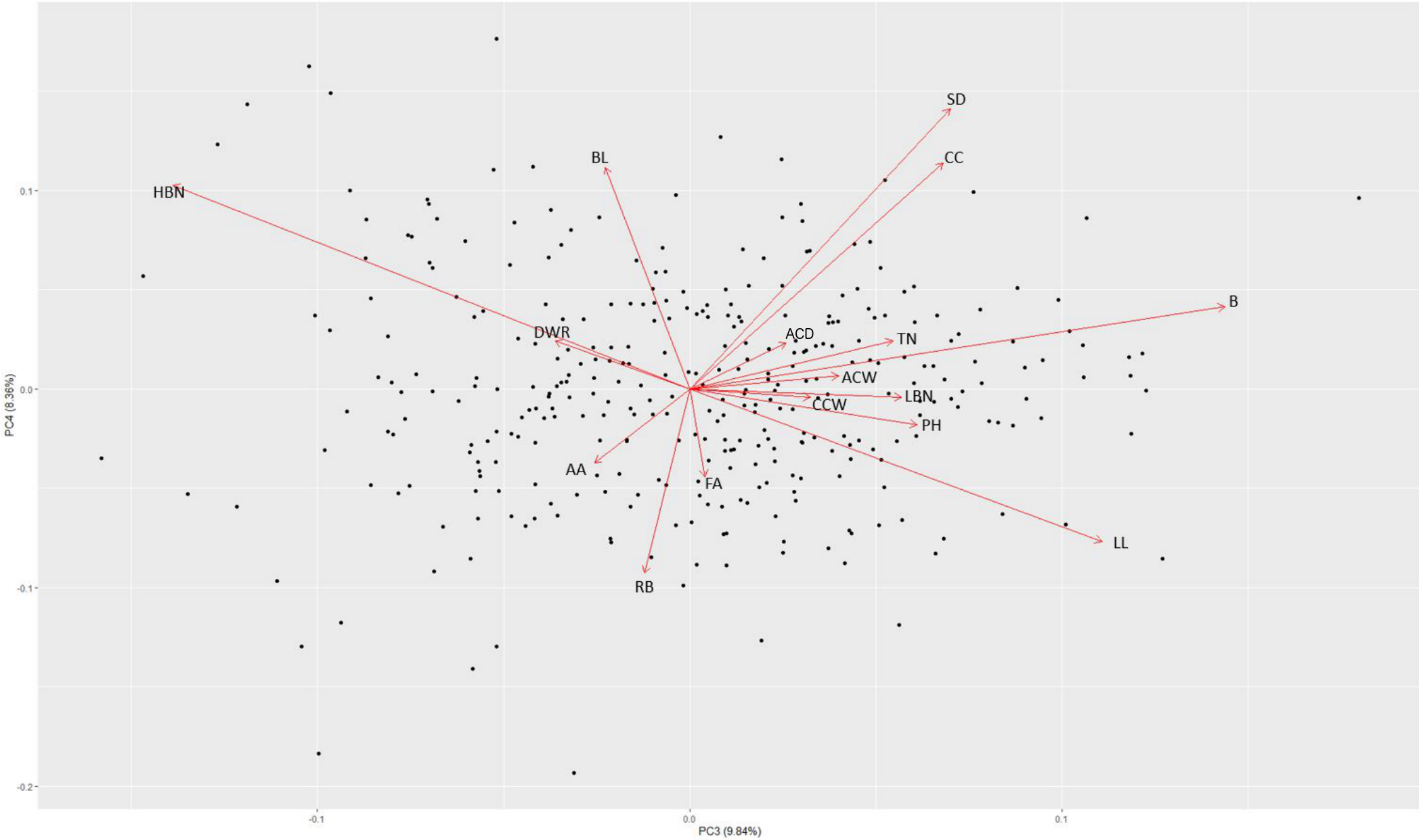

Figure S1: Third and Fourth principal component axes for 340 Maximilian sunflower individuals segregating for apical dominance. FA: First anthesis, AA: average date of anthesis, TN: total nodes, LBN: lowest branching node, RB: reproductive budding, SD: stem diameter, HBN: highest branching node, PH: plant height, ACD: average capitula depth, CCW: central capitulum width, ACW: average capitula width, BL: branch length, LL: leaf length, DWR: capitulum depth:width ratio, B: branches, CC: total capitula count.
