# Supplementary material for: Genetic Analysis of Domestication Parallels in Annual and Perennial Sunflowers (Helianthus spp.): Routes to Crop Development
Source: Front Plant Sci. 2020 Jun 12;11:834. doi: 10.3389/fpls.2020.00834 (PMC7304338; doi:10.3389/fpls.2020.00834)
Supplement: Supplementary file 2 [file Data_Sheet_2.PDF]

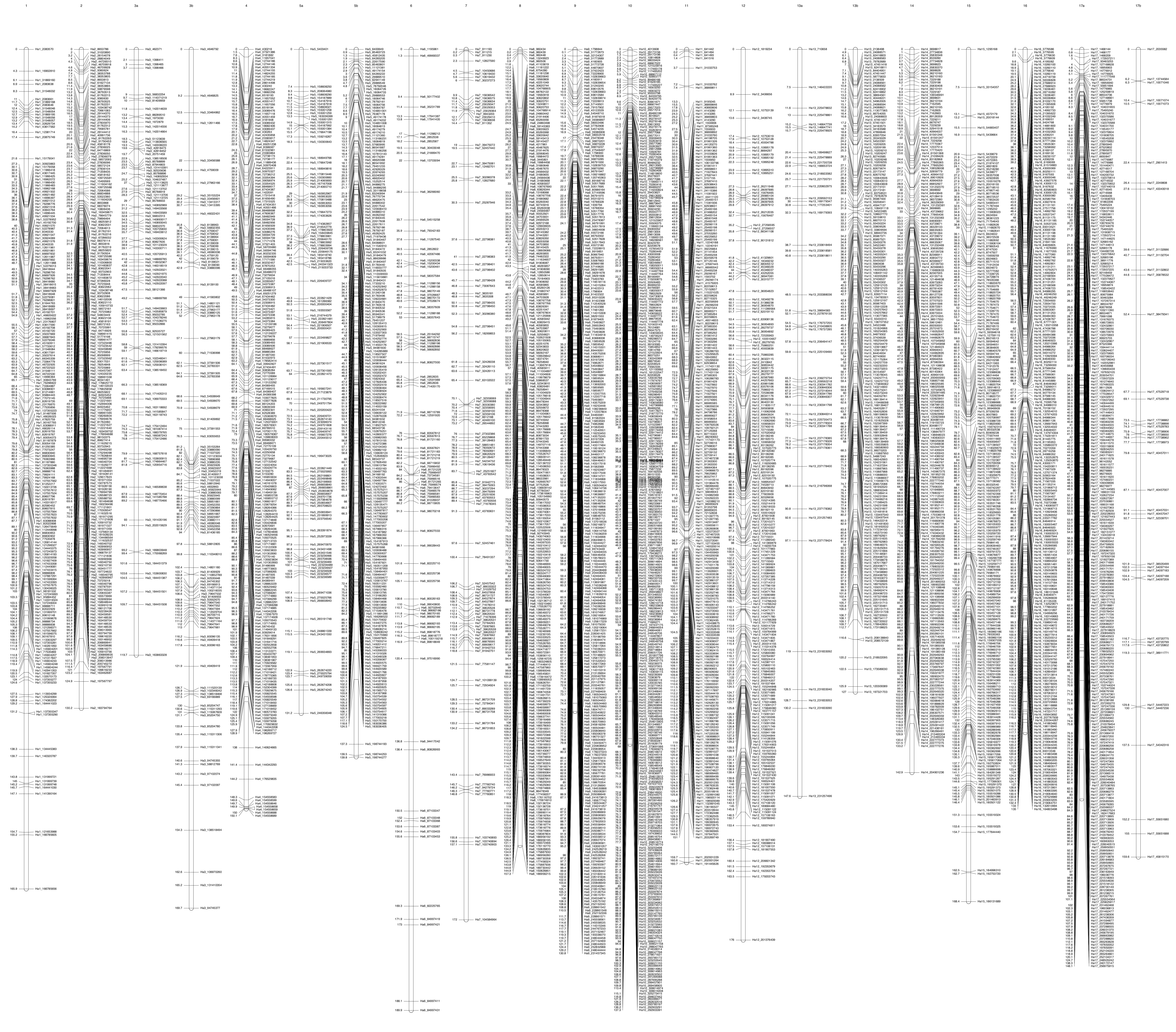


**Figure S2**: Genetic map of Maximilian sunflower generated using 190 F2 individuals ordered in Joinmap 5.0 using the HA412.v.1.1.bronze.20142015 reference genome sequence as a guide.
